# Supplementary material for: Orthostatic Intolerance in Adults Reporting Long COVID Symptoms Was Not Associated With Postural Orthostatic Tachycardia Syndrome
Source: Front Physiol. 2022 Mar 4;13:833650. doi: 10.3389/fphys.2022.833650 (PMC8931464; doi:10.3389/fphys.2022.833650)
Supplement: Supplementary file 1 [file Data_Sheet_1.PDF]

Supplementary Material

1 COVID-19 and non-COVID-19 exclusion criteria for enrolment.

COVID-19-related exclusion criteria were: (1) being in the acute COVID-19 phase and/or experiencing any of the following symptoms/situations included in a COVID-19 Health Screening Assessment Tool administered prior to enrolment: (2) new cough or shortness of breath, fever or chills within the last 48 hours; (3) new loss/distortion/alteration of sense of smell or taste; sore throat/swollen glands; new headaches; vomiting; diarrhoea; (4) positive COVID-19 test in the last 14 days; (5) awaiting a test result for COVID-19; (6) in close contact with someone who had COVID-19 symptoms in the last 14 days; (7) visit to a residential institution in the last 14 days where ongoing COVID-19 transmission had been confirmed; (8) being from a geographical region where there was ongoing COVID-19 transmission and on lockdown/travel restriction; (9) international travel in the last 14 days; or (10) any other symptoms/situations as per Principal Investigator's clinical judgement.

Non-COVID-19 exclusion criteria were: (11) being pregnant; (12) cognitive impairment/dementia with inability to give informed consent; (13) upper limb lymphoedema (cuff-site); and (14) severe skin allergies to adhesive tapes. The following were contra-indications for tilt testing: (15) stroke or acute myocardial infarction within the past 90 days (or any other acute cardiac event); (16) unstable angina; (17) uncontrolled cardiac arrhythmias causing symptoms or hemodynamic compromise; (18) uncontrolled symptomatic heart failure; (19) symptomatic severe aortic stenosis; (20) suspected or known dissecting aortic aneurysm; (21) acute myocarditis or pericarditis; (22) acute pulmonary embolus or pulmonary infarction; (23) patients in whom low organ perfusion pressures may compromise end-artery supplied tissue; (24) severe left ventricular outflow obstruction; and (25) critical mitral stenosis. In addition, for safety reasons, participants who during the anamnesis reported a history of (26) recurrent vasovagal syncope (at least two lifetime episodes) or had a (27) body weight >120 Kg were excluded from tilt testing.

2 Comparison between OI_{tilt} and non-OI_{tilt} subgroups. OI_{tilt}: orthostatic intolerance during tilt; OI_{AS}: orthostatic intolerance during active stand; SD: standard deviation; BMI: body mass index; IQR: interquartile range; CFQ: Chalder Fatigue Scale; CES-D: Center for Epidemiological Studies Depression scale; IES-R: Impact of Event Scale – Revised; cOH_{tilt}: classical orthostatic hypotension during tilt; POTS: postural orthostatic tachycardia syndrome.

Characteristic	No OI_{tilt} (n=43)	OI_{tilt} (n=28)	P
Mean age, years (SD)	47.1 (10.6)	44.1 (9.7)	0.281[a]
Female sex (%)	69.8	75.0	0.632[b]
Third level education (%)	65.9	63.0	0.807[b]
Previous or current smoker (%)	51.3	40.7	0.399[b]
History of hypertension (%)	20.9	14.3	0.479[b]
History of heart disease (%)	4.7	0.0	0.515[c]
History of diabetes (%)	7.0	0.0	0.273[c]
Median days post-COVID-19 diagnosis (IQR)	269.0 (327.0)	234.0 (339.5)	0.767[a]
Hospitalised with COVID-19 (%)	26.8	26.9	0.993[b]
Median CFQ score (IQR)	25.5 (9.8)	25.0 (8.5)	0.994[a]
Median CES-D score (IQR)	19.0 (16.5)	23.0 (19.0)	0.267[a]
Median IES-R score (IQR)	22.0 (31.0)	26.0 (31.0)	0.585[a]
Mean BMI, kg/m^2 (SD)	28.0 (5.4)	28.6 (4.9)	0.410[a]
Mean 5-chair stands time, seconds (SD)	14.8 (10.5)	13.8 (9.3)	0.827[a]
OI_{AS}	51.2	78.6	0.020[b]
cOH_{tilt}	31.0	32.1	0.916[b]
HR increase >30 bpm without OH_{tilt}	14.3	3.7	0.233[c]
On antihypertensive (%)	18.6	14.3	0.753[c]
On beta blocker (%)	9.3	21.4	0.177[c]
On antidepressant (%)	11.6	28.6	0.071[b]
On benzodiazepine (%)	2.3	3.6	0.100[c]

[a] 2-sided Mann-Whitney U test; [b] Chi-square test; [c] 2-sided Fisher's exact test; [*] statistically significant (P<0.05)

3 Haemodynamic comparison between OItilt and non-OItilt subgroups. OItilt: orthostatic intolerance during tilt; SD: standard deviation; SBP: systolic blood pressure; DBP: diastolic blood pressure; HR: heart rate; bpm: beats per minute; TSI: tissue saturation index.

	No OI_{tilt} (initial n=43) (final n=38)	OI_{tilt} (initial n=28) (final n=15)	P
Mean oscillometric baseline SBP, mmHg (SD)	133.0 (11.7) (range 112 – 160)	134.9 (13.7) (range 105 – 162)	0.504[a]
Tilt: mean baseline SBP, mmHg (SD)	130.1 (13.7)	130.7 (11.3)	0.801[a]
Tilt: mean nadir SBP, mmHg (SD)	123.1 (17.3)	121.2 (22.0)	0.666[a]
Tilt: mean SBP at 1 minute, mmHg (SD)	131.6 (15.9)	132.3 (17.0)	0.970[a]
Tilt: mean SBP at 2 minutes, mmHg (SD)	132.3 (19.6)	133.1 (15.9)	0.707[a]
Tilt: mean SBP at 3 minutes, mmHg (SD)	133.0 (18.0)	133.4 (19.6)	0.989[a]
Tilt: mean SBP at 4 minutes, mmHg (SD)	135.7 (20.0)	133.5 (19.9)	0.933[a]
Tilt: mean SBP at 5 minutes, mmHg (SD)	132.2 (17.8)	134.4 (14.5)	0.538[a]
Tilt: mean SBP at 6 minutes, mmHg (SD)	132.6 (22.0)	131.0 (15.6)	0.699[a]
Tilt: mean SBP at 7 minutes, mmHg (SD)	134.6 (20.8)	130.2 (16.8)	0.583[a]
Tilt: mean SBP at 8 minutes, mmHg (SD)	132.1 (16.9)	132.9 (14.7)	0.682[a]
Tilt: mean SBP at 9 minutes, mmHg (SD)	134.3 (16.2)	132.3 (16.2)	0.585[a]
Tilt: mean SBP at 10 minutes, mmHg (SD)	137.9 (17.9)	128.5 (15.9)	0.069[a]
Mean oscillometric baseline DBP, mmHg (SD)	81.2 (9.4) (range 66 – 104)	83.9 (9.8) (range 64 – 99)	0.169[a]
Tilt: mean baseline DBP, mmHg (SD)	78.3 (10.5)	79.4 (9.9)	0.449[a]
Tilt: mean nadir DBP, mmHg (SD)	81.6 (14.3)	82.0 (17.9)	0.635[a]
Tilt: mean DBP at 1 minute, mmHg (SD)	86.0 (11.0)	89.6 (16.3)	0.494[a]
Tilt: mean DBP at 2 minutes, mmHg (SD)	88.1 (14.4)	92.6 (16.9)	0.347[a]
Tilt: mean DBP at 3 minutes, mmHg (SD)	87.0 (12.4)	88.2 (19.1)	0.479[a]
Tilt: mean DBP at 4 minutes, mmHg (SD)	89.0 (14.9)	88.6 (17.9)	0.989[a]
Tilt: mean DBP at 5 minutes, mmHg (SD)	86.6 (13.9)	88.8 (13.6)	0.816[a]
Tilt: mean DBP at 6 minutes, mmHg (SD)	88.1 (14.4)	90.8 (14.4)	0.705[a]
Tilt: mean DBP at 7 minutes, mmHg (SD)	88.5 (15.4)	91.9 (15.4)	0.536[a]
Tilt: mean DBP at 8 minutes, mmHg (SD)	89.0 (12.3)	90.5 (16.6)	0.913[a]
Tilt: mean DBP at 9 minutes, mmHg (SD)	88.9 (13.1)	91.6 (13.7)	0.655[a]
Tilt: mean DBP at 10 minutes, mmHg (SD)	90.9 (15.1)	87.5 (14.3)	0.337[a]
Tilt: mean baseline HR, bpm (SD)	67.4 (10.0) (range 47 – 91)	64.7 (12.3) (range 46 – 95)	0.166[a]
Tilt: mean nadir HR, bpm (SD)	74.7 (15.2)	71.8 (17.0)	0.193[a]
Tilt: mean HR at 1 minute, bpm (SD)	77.6 (14.5)	76.8 (18.4)	0.341[a]
Tilt: mean HR at 2 minutes, bpm (SD)	78.2 (14.3)	77.8 (14.1)	0.812[a]
Tilt: mean HR at 3 minutes, bpm (SD)	78.5 (14.6)	80.5 (18.4)	0.934[a]
Tilt: mean HR at 4 minutes, bpm (SD)	78.6 (13.4)	78.7 (14.3)	0.933[a]
Tilt: mean HR at 5 minutes, bpm (SD)	80.7 (13.6)	80.1 (11.6)	0.942[a]
Tilt: mean HR at 6 minutes, bpm (SD)	80.3 (13.3)	81.2 (19.0)	0.738[a]
Tilt: mean HR at 7 minutes, bpm (SD)	80.6 (14.1)	83.2 (17.8)	0.925[a]
Tilt: mean HR at 8 minutes, bpm (SD)	80.0 (14.2)	80.5 (19.6)	0.548[a]
Tilt: mean HR at 9 minutes, bpm (SD)	81.7 (14.9)	81.2 (18.2)	0.428[a]
Tilt: mean HR at 10 minutes, bpm (SD)	80.3 (14.8)	82.0 (23.0)	0.724[a]
Tilt: mean baseline TSI, % (SD)	70.0 (4.7) (range 56 – 79)	69.5 (4.5) (range 62 – 84)	0.450[a]
Tilt: mean nadir TSI, % (SD)	69.1 (5.1)	69.1 (3.8)	0.793[a]
Tilt: mean TSI at 1 minute, % (SD)	68.9 (4.5)	68.4 (3.7)	0.521[a]
Tilt: mean TSI at 2 minutes, % (SD)	67.9 (5.0)	67.8 (3.3)	0.677[a]
Tilt: mean TSI at 3 minutes, % (SD)	67.9 (5.8)	68.2 (3.4)	0.868[a]
Tilt: mean TSI at 4 minutes, % (SD)	68.1 (6.0)	68.0 (3.5)	0.577[a]
Tilt: mean TSI at 5 minutes, % (SD)	68.0 (6.0)	67.8 (4.1)	0.515[a]
Tilt: mean TSI at 6 minutes, % (SD)	68.4 (5.5)	66.6 (3.1)	0.091[a]
Tilt: mean TSI at 7 minutes, % (SD)	68.5 (5.4)	67.2 (2.9)	0.155[a]
Tilt: mean TSI at 8 minutes, % (SD)	69.0 (5.6)	67.5 (3.2)	0.132[a]
Tilt: mean TSI at 9 minutes, % (SD)	68.7 (5.3)	67.7 (3.1)	0.142[a]
Tilt: mean TSI at 10 minutes, % (SD)	69.3 (4.0)	69.7 (5.9)	0.892[a]

18 of the 71 participants had an early tilt termination (n=2 before the 2nd minute, n=3 before the 3rd minute, n=1 before the 4th minute, n=2 before the 5th minute, n=5 before the 6th minute, n=2 before the 8th minute, and n=3 before the 10th minute). Of all the early terminations, 3 did not relate to symptom development (n=3). [a] 2-sided Mann-Whitney U test; [b] Chi-square test; [*] statistically significant ($P<0.05$). Two-way ANOVA P values for the interaction: SBP: P=0.095; DPB: P=0.280; HR: P=0.494; TSI: P=0.219.

4 Haemodynamic visualisation of OItilt and non-OItilt groups: a: systolic blood pressure (SBP); b: diastolic blood pressure (DBP); c: heart rate (HR); d: tissue saturation index (TSI). CI: confidence interval.

a: systolic blood pressure (SBP)

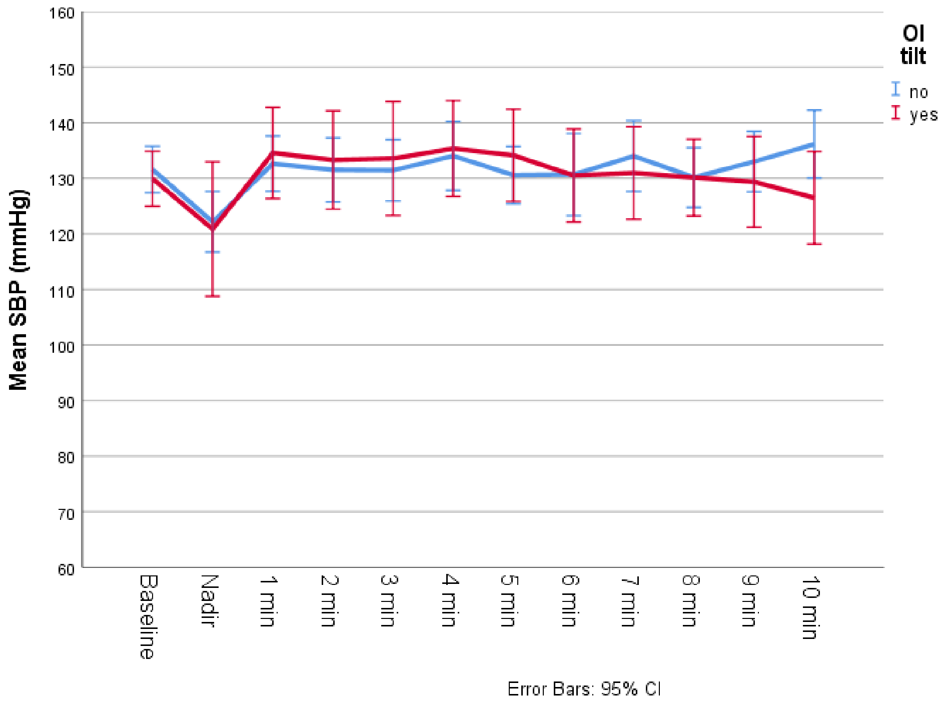

b: diastolic blood pressure (DBP)

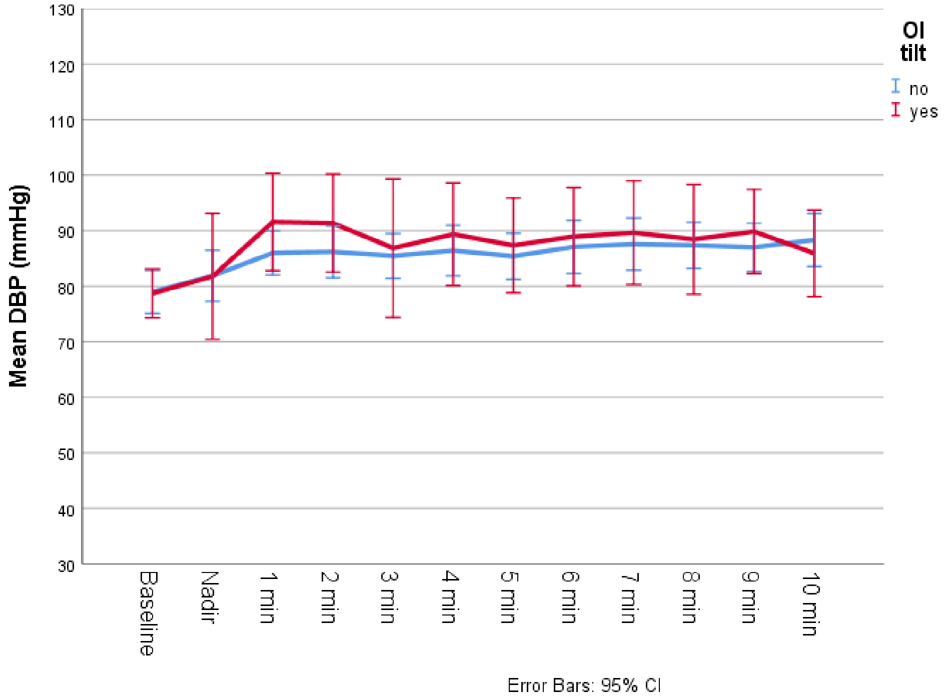

c: heart rate (HR)

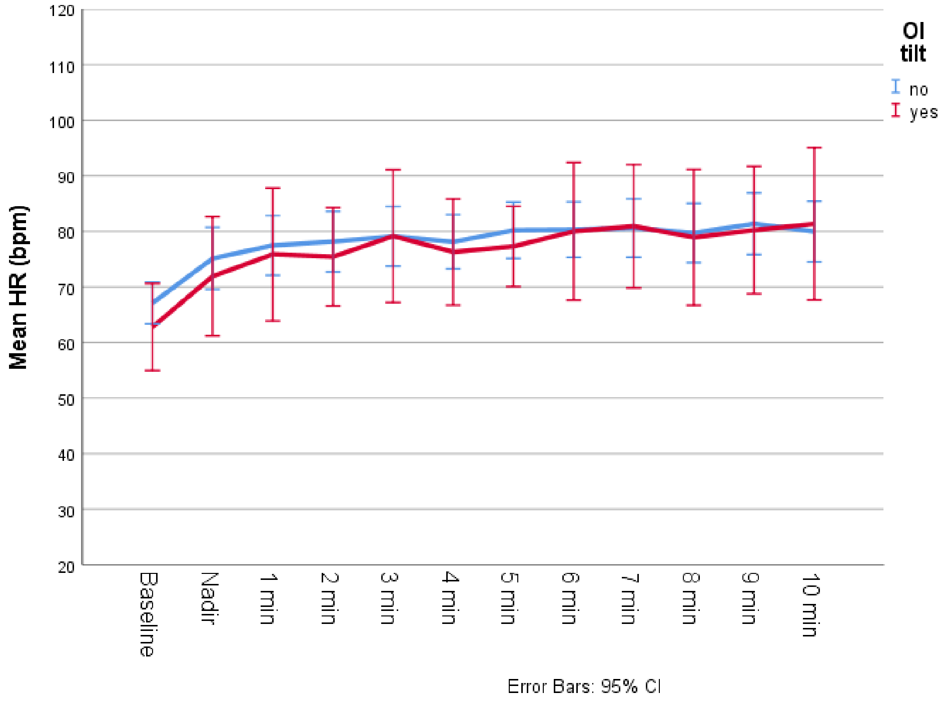

d: tissue saturation index (TSI)

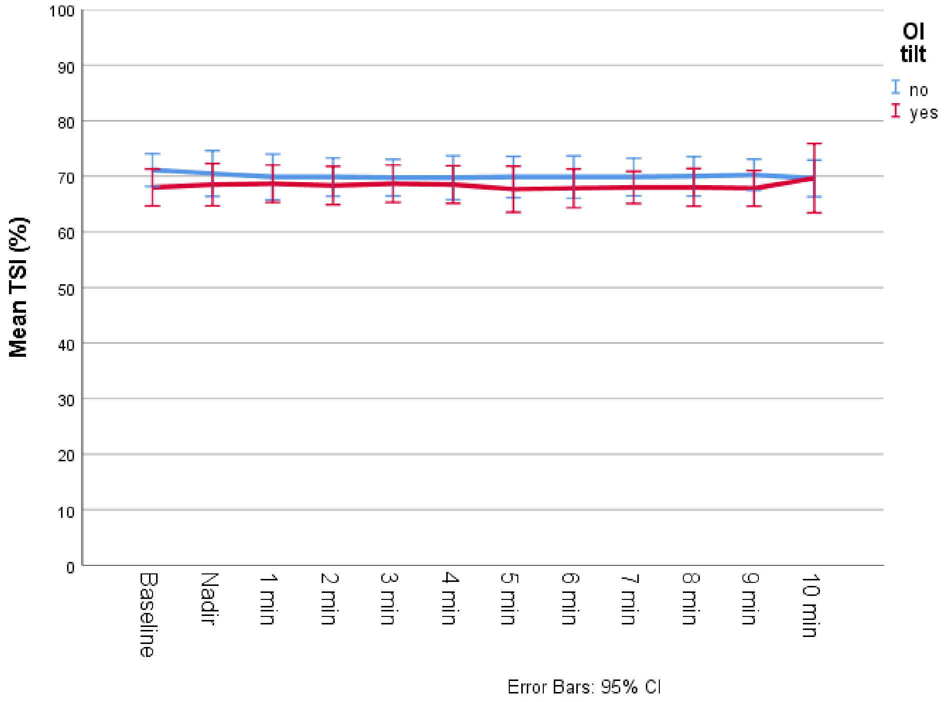

5 Logistic regression model with predictors of OItilt. OItilt: orthostatic intolerance during tilt; CFQ: Chalder Fatigue Scale; CES-D: Center for Epidemiological Studies Depression scale; cOHtilt: classic orthostatic hypotension during tilt; POTS: postural orthostatic tachycardia syndrome; OR: odds ratio; CI: confidence interval.

	OR	95% C.I. for OR		P
		Lower	Upper	
Age	0.96	0.90	1.02	0.202
Female sex	2.99	0.71	12.65	0.136
CFQ score	0.97	0.86	1.08	0.547
CES-D score	1.02	0.97	1.08	0.435
cOH_{tilt}	1.04	0.28	3.79	0.955
HR increase >30 bpm without OH_{tilt}	0.15	0.01	1.53	0.109
Lowest SBP after tilt	1.00	0.97	1.04	0.858
